# Effect of questionnaire length, personalisation and reminder type on response rate to a complex postal survey: randomised controlled trial

**DOI:** 10.1186/1471-2288-11-62

**Published:** 2011-05-06

**Authors:** Shannon Sahlqvist, Yena Song, Fiona Bull, Emma Adams, John Preston, David Ogilvie

**Affiliations:** 1 Medical Research Council Epidemiology Unit and UKCRC Centre for Diet and Activity Research (CEDAR), Box 296, Institute of Public Health, Forvie Site, Robinson Way, Cambridge, CB2 0SR, UK; 2 Transportation Research Group, School of Civil Engineering and the Environment, University of Southampton, Highfield, Southampton, SO17 IBJ, UK; 3 School of Sport, Exercise and Health Sciences, Loughborough University, Ashby Rd, Loughborough, LE13 3TU, UK; 4 School of Population Health, University of Western Australia, 35 Stirling Highway, Crawley, WA, 6009, Australia

## Abstract

**Background:**

Minimising participant non-response in postal surveys helps to maximise the generalisability of the inferences made from the data collected. The aim of this study was to examine the effect of questionnaire length, personalisation and reminder type on postal survey response rate and quality and to compare the cost-effectiveness of the alternative survey strategies.

**Methods:**

In a pilot study for a population study of travel behaviour, physical activity and the environment, 1000 participants sampled from the UK edited electoral register were randomly allocated using a 2 × 2 factorial design to receive one of four survey packs: a personally addressed long (24 page) questionnaire pack, a personally addressed short (15 page) questionnaire pack, a non-personally addressed long questionnaire pack or a non-personally addressed short questionnaire pack. Those who did not return a questionnaire were stratified by initial randomisation group and further randomised to receive either a full reminder pack or a reminder postcard. The effects of the survey design factors on response were examined using multivariate logistic regression.

**Results:**

An overall response rate of 17% was achieved. Participants who received the short version of the questionnaire were more likely to respond (OR = 1.48, 95% CI 1.06 to 2.07). In those participants who received a reminder, personalisation of the survey pack and reminder also increased the odds of response (OR = 1.44, 95% CI 1.01 to 1.95). Item non-response was relatively low, but was significantly higher in the long questionnaire than the short (9.8% vs 5.8%; *p *= .04). The cost per additional usable questionnaire returned of issuing the reminder packs was £23.1 compared with £11.3 for the reminder postcards.

**Conclusions:**

In contrast to some previous studies of shorter questionnaires, this trial found that shortening a relatively lengthy questionnaire significantly increased the response. Researchers should consider the trade off between the value of additional questions and a larger sample. If low response rates are expected, personalisation may be an important strategy to apply. Sending a full reminder pack to non-respondents appears a worthwhile, albeit more costly, strategy.

## Background

Postal surveys are widely used in public health research as they provide a low cost, efficient and relatively unobtrusive way to reach large numbers of people [1,2]. Their use, however, is associated with several limitations. Participant, or unit, non-response is common and can affect the external validity of the findings [1]. As postal surveys are self-administered, item non-response - resulting from either the layout of the questionnaire or participants' reluctance to disclose certain information - can also occur, affecting the internal validity and utility of the data [3,4].

A recent meta-analysis suggests that a number of strategies can maximise participant response [5]. These include, but are not limited to, providing incentives, pre-notifying participants, developing an appealing survey pack, personally addressing the survey pack and following up (reminding) non-respondents [5]. The nature of the follow-up appears to be important in that sending a second copy of the survey pack is more beneficial than sending a reminder notification only [1]. Sending a second survey pack is, however, more costly, and the benefits of increased participation need to be traded off against the greater costs incurred. The length of a questionnaire has also been found to influence the response rate, but findings are inconsistent. Earlier studies suggest that response rates decrease once length exceeds 12 pages [2], while more recent research suggests no effect of length when the questionnaire is over 4 pages long [6]. For example, Mond and colleagues [7] reported no difference in response rate between an 8- and a 14-page questionnaire on eating disorders that was hand delivered to women at home.

The applicability of this body of evidence to public health is limited. Much of it derives from the fields of marketing and education [1], and research in the health field has generally focused on the health care setting and specific target groups such as doctors and patients [8] rather than the population at large. Moreover, a great deal of the research was conducted prior to 2000 and it is likely that the public's reaction to postal surveys, and the influences on participation, have changed over the past decade with increased concerns about privacy, the emergence of new information technologies and the increasing proliferation of unsolicited (junk) mail.

As well as minimising both unit and item non-response, it is also important that the survey sample is representative of the population under investigation. An appropriate sampling frame therefore needs to be selected. In the UK, one of the most commonly used sampling frames for postal surveys is the edited electoral register (ER). The ER lists the name and address of everyone in the UK who has registered to vote. Since 2002, however, electors have been able to opt out of the edited version of the register so that their information is not made available to third parties. Concerned about the impact that that this may have had on the representativeness of the edited ER, the National Centre for Social Research (NatCen), in collaboration with the Office for National Statistics (ONS), assessed the characteristics of adults not listed on the edited ER by comparing the register with households that took part in the ONS Omnibus Survey between April and June 2005. 43% of adults found at the responding addresses were not listed on the edited ER [9]. Those not listed were more likely to be 18 to 24 years of age, renting their accommodation, and to have a university degree [9]. Members of minority ethnic groups were also less likely to be listed [9]. These findings suggest that the edited ER may not be representative of the general population and that alternative sampling frames for population based research should also be considered. One of these is the Postcode Address File (PAF), a list of all mail delivery points in the UK. The PAF does not include residents' names, and as a consequence postal surveys cannot be personally addressed to households sampled from the PAF. This is potentially detrimental as some studies have found that lack of personalisation may affect the response rate to mailed questionnaires [10].

Given the lack of recent, applicable evidence on the influences on population based postal survey participation and concerns about the representativeness of the edited ER, the aim of this randomised controlled trial was to examine the impact of three survey design factors - personalisation, questionnaire length and the nature of the reminder - on unit and item non-response and to compare the cost-effectiveness of the alternative strategies.

## Methods

### Study context

This study was conducted to inform the design of the survey fieldwork for iConnect, a large UK-wide project that aims to examine the impact of infrastructural improvements for walking and cycling on travel behaviour, physical activity and carbon emissions [11]. The infrastructural improvements are the result of Sustrans' Connect2 initiative, which comprises a series of projects to build or improve local walking and cycling routes in 79 communities throughout the UK http://www.sustransconnect2.org.uk. The iConnect research consortium has selected several of these projects for in-depth investigation drawing on an applied ecological evaluation framework [11]. The core research method involves a postal survey administered to a cohort of randomly selected local residents at these sites.

Interdisciplinary evaluative research of this kind involves attempting to measure and characterise a variety of complex behaviours and their putative correlates in a variety of domains. Concerns were raised over the length of the iConnect pilot questionnaire developed to address these measurement aims, and a decision was therefore made to develop and test a second, shorter version of the questionnaire. As with most population based studies, obtaining a representative sample was considered important for the evaluation of the Connect2 projects. To that end, the trial also sought to compare the response obtained by sending a personally addressed survey pack (which is possible using the edited ER, but not using the PAF) with that obtained by sending a survey pack that was not personally addressed.

### Study design and participants

**Figure 1:**
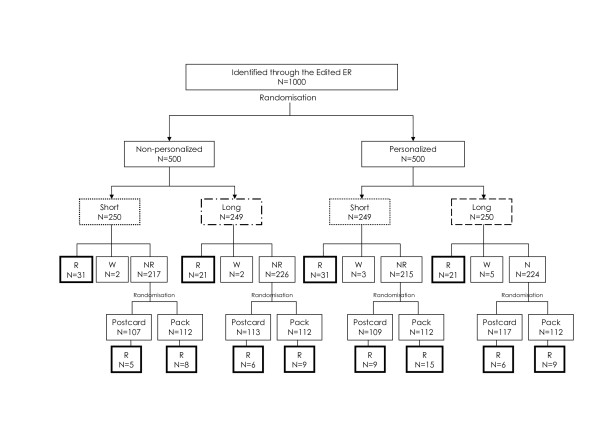
**Participant flow through the study and questionnaire response**. WR: Withdrawn; R: Responded; NR: Not responded.

A 2 × 2 factorial design was used whereby 1000 participants were randomly selected from the UK edited ER. Using a computer generated randomisation sequence participants were allocated to receive either: (a) a personally addressed pack containing a copy of the long questionnaire; (b) a personally addressed pack containing a copy of the short questionnaire; (c) a non-personalised pack containing a copy of the long questionnaire; or (d) a non-personalised pack containing a copy of the short questionnaire (Figure 1). Those who did not return a questionnaire within two weeks were stratified by questionnaire length and approach and further randomised to receive either a reminder postcard or a reminder pack. The reminder pack contained a letter and a second copy of the questionnaire. To examine and control for the possible influence of residential location and socioeconomic status on survey response, four separate electoral wards were selected for sampling: one relatively deprived and one relatively affluent ward identified using the Index of Multiple Deprivation (IMD) from each of two cities, Cambridge and Southampton. Participants were blind to their allocation status and to the fact that these survey design factors were the subject of a randomised controlled trial.

The sample size required to detect a significant difference between two proportions is greatest when one of the proportions is 0.5. In calculating the required sample size, we therefore made the most conservative assumption of a response rate of 50% for the design factor under investigation and a response rate of 40% for the comparison design factor. Specifying an alpha level of .05 and a power of 80%, 816 participants were required to detect a difference of ten percentage points even in the 'worst case' of one of the proportions being as high as 0.5.

### Questionnaires

The long questionnaire was 24 A4 pages and consisted of seven sections (see Additional File). Questions were included to assess perceptions of the neighbourhood [12] and route affected by the intervention and constructs derived from the Theory of Planned Behaviour (TPB) [13]. Travel behaviour was assessed using both a seven-day recall and a more detailed one-day recall instrument. Physical activity was assessed using the Recent Physical Activity Questionnaire (RPAQ) [14], which assesses domain specific physical activity in detail over the previous four weeks.

The short questionnaire covered the same general constructs but was reduced to six sections and 15 A4 pages. The seven-day travel instrument was omitted, items to assess perceptions of the environment and TPB constructs were reduced, and the short form of the International Physical Activity Questionnaire (IPAQ) [15] replaced the RPAQ. Detailed comparison of the two questionnaires can be found in the Additional File.

### Procedures

Several evidence based strategies were used to maximise the response rate. All participants received a forewarning postcard encouraging them to complete the questionnaire. One week later, participants were sent the survey pack which contained a letter of invitation, an information sheet, a consent form, a questionnaire and a freepost return envelope. Participants who did not return their questionnaire within two weeks were sent either a reminder postcard or a reminder pack depending on their randomisation status. Respondents were entered into a prize draw to win one of twenty £25 multi-store gift vouchers on receipt of a completed questionnaire, and a postcard was sent to all respondents thanking them for their participation. The study coordinators charged with receipting the return of completed surveys were not aware of a respondent's allocation status in terms of personalisation and reminder type. Nonetheless, they could not be fully blinded to a respondents allocation status due to the different lengths (and therefore weights) of the two questionnaires. The researcher who conducted the analysis was not involved in the receipt or scrutiny of the questionnaires.

### Analysis

#### Influences on response rate

Questionnaires were visually scanned on receipt. A questionnaire was considered 'usable' if any part of it had been attempted, while receipt of a completely blank questionnaire was recorded as a non-response. Twenty-two participants did not return a signed consent form with their questionnaire, but for the purposes of this analysis the completeness of their survey response was assessed solely on the basis of the questionnaire and not on the completion of the consent form. Response rate was defined as the number of usable returned questionnaires expressed as a percentage of the issued sample. Three outcome measures of response were derived: (1) overall survey response rate, (2) survey response rate prior to reminder and (3) survey response rate only in those who received a reminder. A series of multivariate logistic regression analyses were conducted to examine the influences on response using the three outcome measures. All possible influences (questionnaire length, personalisation, nature of reminder, city, and area level deprivation) were entered into the model to determine their independent effects on survey response.

#### Item non-response

Item non-response was assessed using an established method [16]. The number of missing responses was divided by the total number of items for the entire questionnaire and for each section. Responses that were considered implausible, or that were entered in the wrong format (e.g. multiple responses where only one was required, or free text responses to closed-response questions) were treated as missing. Two-tailed unpaired t-tests (assuming different standard deviations between groups) were conducted to assess the statistical significance of the differences observed.

#### Cost-effectiveness

The total cost of each survey pack was determined by summing the cost of printing, packing, and posting (but not returning) all relevant materials. Staff costs related to tracking the returned questionnaires and answering respondents' queries were not included. Cost-effectiveness was defined as the cost incurred per returned usable questionnaire in each arm of the trial.

Data were analysed using SPSS for Windows (Version 16.0, 2004, SPSS Inc., Chicago, USA).

## Results

**Figure 2:**
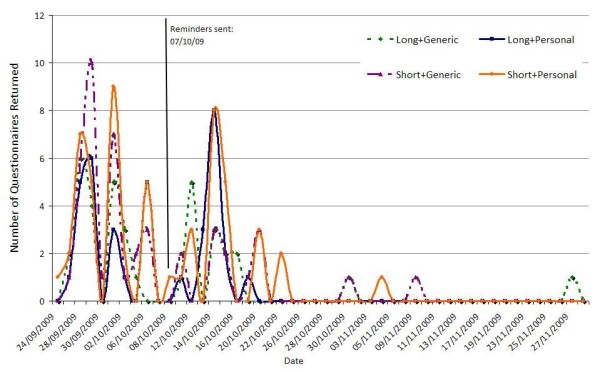
**Time course of return of questionnaires**.

The pattern of survey response is summarised in Figure 2. Of the 1000 participants who received a forewarning postcard, two formally withdrew from the study and were not sent a survey pack. Overall, 171 questionnaires were returned - a response rate of 17%. 91 participants returned a completed questionnaire (and 12 returned a blank questionnaire) before the reminder was mailed, leaving 895 participants to be randomly allocated to receive either a reminder postcard (n = 447) or reminder pack (n = 448). Of these, 13 participants returned a completed questionnaire before the reminder could have been influential (i.e. within two days of the reminder being sent) and were therefore included in the calculation of questionnaire response prior to reminder.

Questionnaire response rate prior to reminder was 10% (n = 104); an additional 7% (n = 67) were returned after the reminder. Overall, 18% (n = 91; 52 before and 39 after reminder) of the personalised questionnaires were returned compared with 16% (n = 80; 52 before and 28 after reminder) of the non-personalised questionnaires. 20% (n = 99) of the short questionnaires were returned compared with 14% (n = 72) of the long questionnaires. Among those classified as having returned the questionnaire after receiving a reminder (n = 889), 9% (n = 41) of those who received a reminder pack returned the questionnaire compared with 6% (n = 26) of those who received a reminder postcard.

### Effect of survey design factors on response rate

In multivariate logistic regression analyses, of the three survey design factors examined, questionnaire length had the strongest independent influence on response rate: the odds of response were approximately 50% higher for the short version of the questionnaire compared with the long version (OR 1.48, 95% CI 1.06 to 2.07: Table 1). Personally addressing the survey pack did not significantly influence the overall response rate (OR 1.17, 95% CI 0.84 to 1.64), but among participants who received a reminder, the use of a personally addressed reminder increased the odds of response by nearly half (OR 1.44, 95% CI 1.01 to 1.95). Among participants who received a reminder, those who received a second full survey pack were almost 40% more likely to respond than those who received only a reminder postcard, but this difference was not statistically significant (OR 0.62, 95% CI 0.37 to 1.04). Area level deprivation showed a strong negative association with response rate, participants living in the two relatively deprived wards being 50% less likely to respond than those in the two relatively affluent wards (OR 0.52, 95% CI 0.37 to 0.73). There was no significant difference in response between residents of the two cities.

**Table 1:** Adjusted odds ratios and 95% CI for survey response overall, prior to reminder, and after reminder

	Overall^a^ N = 1000		Prior to reminder^b^ N = 1000		After reminder^c^ N = 882	
	OR	95% CI	OR	95% CI	OR	95% CI
Length						
Long	1.00		1.00		1.00	
Short	1.48*	1.06-2.07	1.62*	1.07-2.45	1.32	0.79-2.18
Reminder						
Pack	-	-	-	-	1.00	
Postcard	-	-	-	-	0.62	0.37-1.04
Approach						
Non-personal	1.00		1.00		1.00	
Personal	1.17	0.84-1.64	1.00	0.67-1.50	1.44*	1.01-1.95
Index of Multiple Deprivation						
Relatively affluent	1.00		1.00		1.00	
Relatively deprived	0.52*	0.37-0.73	0.65*	0.43-0.98	0.44*	0.26-0.75
City						
Cambridge	1.00		1.00		1.00	
Southampton	0.90	0.65-1.26	0.96	0.64-1.43	0.78	0.54-1.49

OR = Odds ratio; 95%CI: 95% confidence interval, * = *p *< .05; ORs are adjusted for all other covariates listed in the table.

**^a^**Hosmer & Lemeshow test χ^2 ^= 7.79, df = 6, *p *= .25; Nagelkerke's R^2 ^= 3.5%

**^b^**Hosmer & Lemeshow test χ^2 ^= 3.52, df = 5, *p *= .79; Nagelkerke's R^2 ^= 1.9%

**^c^**Hosmer & Lemshow test χ^2 ^= 6.13, df = 8, *p *= .63; Nagelkerke's R^2 ^= 4.4%

### Item non-response

Overall, 8.4% of items were not answered. In both questionnaires, approximately 4% of item non-response was attributable to implausible answers. Item non-response was significantly higher in the long questionnaire (9.8%) than in the short questionnaire (5.8%) (p = 0.04: Table 2). Section A had the lowest item non-response. In the long questionnaire, section F, which included the recreational activity component of the RPAQ, had the highest item non-response. Section C, which contained the questions on travel behaviour, also had a relatively high item non-response. This was also the section with the highest frequency of item non-response in the short questionnaire.

**Table 2:** Item non-response by questionnaire length

Section	**Item non-response **(%)		*p*
	Long	Short	
Overall	9.8	5.8	**.04**
A. Your neighbourhood and local area	2.8	2.5	.73
B. Walking and cycling	12.9	4.9	**.01**
C. Your travel	12.5	9.5	.27
D. Activities at home	6.5	n/a	-
E. Activities at work or place of study	12.4	8.6	.35
F. Recreational activities	15.1	8.3	.11
G. You and your household	6.0	9.6	**.05**

### Cost-effectiveness

The cost per returned questionnaire was £40.7 for the long questionnaire compared with £22.4 for the short questionnaire, a difference of approximately £18 per returned questionnaire (Table 3). For the reminder pack, the additional cost per returned questionnaire was £23.1 (averaged across the long and short questionnaires) compared with an additional £11.3 for the reminder postcard.

**Table 3:** Cost-effectiveness of postal survey approaches

	**Long**			**Short**			**Combined**		
	
	**Survey pack**	**Reminder^b^**		**Survey pack**	**Reminder^b^**		**Survey pack**	**Reminder^b^**	
						
		**Card**	**Pack**		**Card**	**Pack**		**Card**	**Pack**
	
No. printed	500	231	216	500	224	224	1000	455	440
Total cost (£)	1712.8	150.1	545.1	1385.6	144.4	402.6	3098.4	294.5	947.7
Unit cost (£)	3.4	0.7	2.5	2.8	0.6	1.8	4.8	0.6	2.2
No. returned	42	12	18	62	14	23	104	26	41
Cost-effectiveness^a ^(£)	40.7	12.5	30.3	22.4	10.3	17.5	29.8	11.3	23.1

^a ^Cost per usable survey returned

^b ^Additional cost of reminder

## Discussion

This study examined the impact of several strategies on postal survey response rate. Response quality and cost-effectiveness were also examined. Overall, a response rate of 17% was achieved. Adult participants who received the short version of the questionnaire, and those living in the relatively affluent electoral wards, were approximately 50% more likely to respond than those who received the long version of the questionnaire and those living in the relatively deprived wards respectively. Encouragingly, item non-response was relatively low in both questionnaires, but as expected was higher in the long questionnaire.

Several strategies were used to maximise the response rate, including pre-notifying participants of the survey and entering respondents into a prize draw. Nonetheless, the overall response rate was low. This response rate is comparable to that obtained in another similar postal survey [17], although two other recent surveys have reported response rates of 70% and 33% respectively [18,19]. All three studies were similar to ours in that they included questions on physical activity behaviour, attitudes towards physical activity and perceptions of the neighbourhood environment. However, our questionnaire also included detailed questions on travel behaviour which required participants to recall the travel modes, durations and distances of all journeys taken. These complex but important questions may have deterred participation. A recent study conducted in Glasgow, Scotland, using comparable procedures - albeit with a more deprived population - included measures of travel and physical activity behaviour and obtained a similar response rate of 15% [20]. The low response rate achieved in this and other studies [17,20,21] may also reflect a more general downward trend in participation in population surveys irrespective of the mode of data collection [4,22,23].

Our findings contradict previous reports that above a relatively low threshold, questionnaire length has no influence on unit non response [7,24]. Of all the survey design factors examined in this trial, questionnaire length was the most influential. This finding may be partly explained by the length of the questionnaires tested, both of which were longer than those issued in most previous studies of the influence of length on response. A study following up mental health patients examined questionnaires of comparable length to those used in our study and found that a 13-page questionnaire elicited a greater response than a 23-page questionnaire [25]. Questionnaires of the length used in our study are typical in the fields of travel and physical activity research, as the behaviours of interest are complex and difficult to assess with only one or two items. Our findings suggest that when developing questionnaires of this nature, there is value in reducing the length of the questionnaire as an increase in length may reduce the response rate.

In developing the short questionnaire we omitted a detailed instrument assessing travel behaviour in the previous seven days and substituted a comprehensive measure of physical activity (RPAQ) with a shorter measure with poorer validity (IPAQ). We considered that this loss in detail regarding travel and physical activity behaviour was adequately compensated for by the increased response rate achieved. Researchers should remain mindful of questionnaire length and carefully consider the trade off between the value of additional questions and the value of a larger sample.

As well as influencing unit non-response, questionnaire length also influenced item-non response which overall, was 5.8% for the short questionnaire compared with 9.8% for the long questionnaire. Although counterintuitive, our findings indicate that item non-response to the demographic questions (Section G) was higher in the short questionnaire. It could be that the when completing a longer questionnaire respondents are more likely to become desensitised to answering personal questions.

In those participants who received a reminder, personally addressing the survey pack and the subsequent reminder increased the odds of response by an estimated 44%. Given that almost 90% of the sample in this study required a reminder, personalisation may be an important strategy to apply if low response rates are expected, particularly if multiple reminders might be used (although the use of multiple reminders was not investigated in the current study). However, the effect of personalisation as a whole was a non-significant 20% increase in the odds of response, a finding which is consistent with previous research [10]. Despite the 20% increase in response, it seems important to be aware of the self-selection biases that may apply to sampling frames such as the edited ER from which a personalised mailing list can be derived.

In this study, sending a reminder pack increased the odds of response by 60% compared with sending only a reminder postcard. In light of the low response rate, sending a reminder pack therefore appears a worthwhile strategy, although the cost per returned questionnaire was £11.8 higher than when a reminder postcard was used. Where funds are limited, a reminder postcard appears to be an efficient, albeit less effective, method of increasing participant response.

A key strength of this study was that participants were randomly allocated to study arms, which ensures high internal validity. The study was purposely conducted during September and October, a time of year when UK residents are more likely to be at home (as opposed to away on holiday) and when extreme weather patterns are unlikely to influence their travel behaviour. There was, however, a national postal strike during the period of data collection which caused considerable delays in delivery and resulted in a backlog of undelivered mail which may have undermined the response rate. The measure of PA used in the short questionnaire was different to that which was used in the long questionnaire. Subsequently the difference in unit and item non response between the two questionnaires may also have been due to the fact that different questions were included, and not solely due to the length of the questionnaires. Another limitation of the study is that the ER does not include demographic information, so the extent to which respondents were representative of those sampled could not readily be determined. Finally, to control for the possible influence of socioeconomic status on survey response we selected two relatively deprived and two relatively affluent wards, therefore the response rate achieved from sampling from wards in the middle of the socioeconomic spectrum is unknown.

## Conclusions

This randomised controlled trial examined the relative influence of three survey design factors in maximising participation in a population-based postal survey. Shortening the questionnaire was found to be effective in increasing the response rate. Personalising the survey and issuing full reminder packs may also contribute to this goal. Despite the low overall response rate achieved in this and other recent studies, postal surveys remain an efficient way of collecting information from populations, particularly when the complex nature and length of questions precludes the use of a telephone survey as a realistic option. In light of the general downward trend in survey participation, however, more creative ways of maximising response rates may be increasingly necessary.

## Ethical approval

Ethical approval for this study was obtained from the University of Southampton Ethics Committee (reference number CEE 200809-15).

## Competing interests

The authors declare that they have no competing interests.

## Authors' contributions

SS led the analysis and the writing of the manuscript. YS assisted with data collection, trial administration and analysis. All authors contributed to the design of the study, interpretation of the findings, and critical revision of the manuscript and approved the final version.

## Pre-publication history

The pre-publication history for this paper can be accessed here:

http://www.biomedcentral.com/1471-2288/11/62/prepub

## Supplementary Material

Additional file 1 **Overview and comparison of items included in the long and short versions of the questionnaires**. Description of data: Table detailing the items included in the questionnaires. Click here for file
