# Supplementary material for: Effect of questionnaire length, personalisation and reminder type on response rate to a complex postal survey: randomised controlled trial
Source: BMC Med Res Methodol. 2011 May 6;11:62. doi: 10.1186/1471-2288-11-62 (PMC3110121; doi:10.1186/1471-2288-11-62)
Supplement: Additional file 1 — Overview and comparison of items included in the long and short versions of the questionnaires. Description of data: Table detailing the items included in the questionnaires. [file 1471-2288-11-62-S1.PDF]

Additional File

Overview of items included in the long and short versions of the questionnaires

Section	Topic	Details	Long	Short
**Perceptions of the environment**				
**A**	Perceptions of the neighbourhood environment	Items based on the ALPHA questionnaire [24]	33 Likert scale items<br>▪ Type of residence (4 items)<br>▪ Distance to facilities (8 items)<br>▪ Neighbourhood walking and cycling infrastructure (4 items)<br>▪ Maintenance of infrastructure (3 items)<br>▪ Neighbourhood safety (6 items)<br>▪ Neighbourhood attractiveness (4 items)<br>▪ Walking and cycling connectivity (4 items)	23 items<br>▪ Type of residence (4 items)<br>▪ Distance to facilities (8 items)<br>▪ Neighbourhood walking and cycling infrastructure (2 items)<br>▪ Maintenance of infrastructure (2 items)<br>▪ Neighbourhood safety (5 items)<br>▪ Neighbourhood attractiveness (2 items)
	Route-specific questions	Usual travel mode	1 item<br>Usual mode of travel between home or workplace and city	Remained unchanged
		Purpose of travel	1 item<br>What are your main reasons for travelling between these destinations?	Remained unchanged
		Perceptions of the route<br>Adapted from	11 Likert scale items	Reduced to 7 items

		ALPHA [24]		
**Attitudes towards walking and cycling**				
**B**	Intra-individual constructs	Based on constructs of TPB [11]<br>All questions asked separately for walking for recreation, walking for utility, cycling for recreation and cycling for utility	Habit (2 items)<br>Enjoyment (2 items)<br>Subjective norm (2 items)<br>Behavioural control (2 items)<br>Attitude (2 items)<br>Social norms (1 item)<br>Intention (2 items)	Habit (1 item)<br>Intention (1 item)
**Travel**				
**C**	Vehicle access	No. of vehicles in household	1 item	Remained unchanged
	Cars in household	Type of cars in household	1 item<br>Information on vehicle road tax, fuel type, engine size, age for up to four vehicles	Remained unchanged
	Travel behaviour	1-day recall	For each journey made the previous day:<br>Purpose; origin and destination; start and end time; modes used ; time (min) spent travelling and the distance (miles) travelled for each mode; no. of companion travellers	Remained unchanged
		7-day recall	Record no. of trips made for six trip purposes (to and from work; to and from school or college; in the course of work or employer’s business; shopping,	Omitted

			leisure or visiting friends or relatives; personal business), the mode used (walking, cycling, bus or coach, rail or underground, car, taxi or other) for each of these trip purposes, and the total time (min) spent in each mode for each trip purpose	
**Physical activity**				
**D**	Activities at home	RPAQ	Average time spent watching TV, DVD or video<br>Average time spent using a computer<br>Average no. times climbed stairs	Omitted
**E**	Activity at work or place of study	RPAQ	Employment status<br>Occupation<br>Travel to and from work (distance to work, trip time, trip frequency)<br>Work postcode<br>Facilities at work (8 items; yes/no)	Remained unchanged
**F**	Recreational activity	RPAQ	Record no. of sessions of recreational activities (7 options ranging from 'none' to 'every day'<br>Record average time (minutes) per session for each activity	Replaced with IPAQ [13]<br>Past 7-day recall of PA<br>No. of days and time (minutes) spent in moderate-, vigorous-intensity PA and walking<br>Numbers of hours spent sitting per day
**Demographics**				
**G**	Demographics	Standard	Sex, date of birth, height, weight, household composition, health, ethnicity, income, educational attainment, postcode, years living in current home	Remain unchanged
